# CFD-DEM Fluidized Bed Drying Study Using a Coarse-Graining Technique

**DOI:** 10.1021/acs.iecr.3c02960

**Published:** 2023-11-22

**Authors:** M. J. A. de Munck, E. A. J. F. Peters, J. A. M. Kuipers

**Affiliations:** Multiphase Reactors Group, Department of Chemical Engineering and Chemistry, Eindhoven University of Technology, P.O. Box 513, Eindhoven 5600 MB, The Netherlands

## Abstract

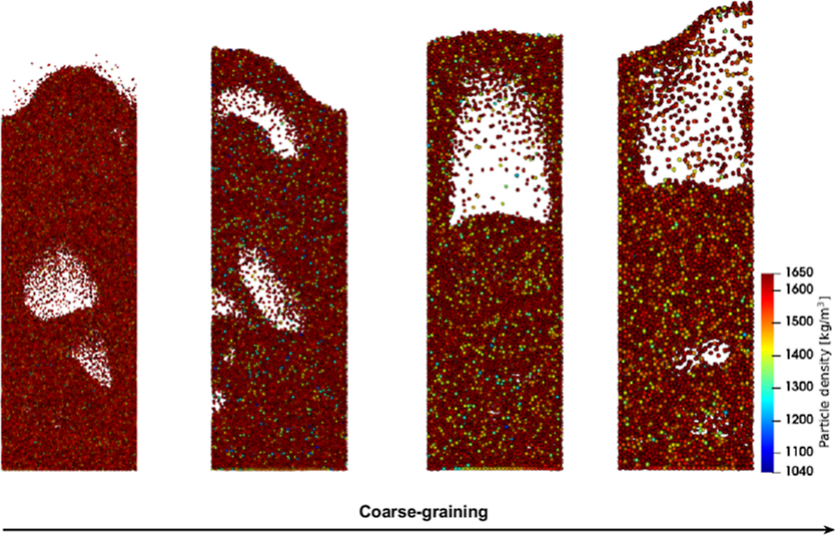

Fluidized beds are commonly applied to industrial drying applications. Modeling using the computational fluid dynamics-discrete element method (CFD-DEM) can be employed to increase the fundamental understanding of solids drying. A large drawback of CFD-DEM is the computational requirements, leading to a limitation regarding the system size. Coarse-grained CFD-DEM is an approach to reduce computational costs, allowing one to simulate larger fluidized beds. In this article, coarse-graining CFD-DEM scaling laws are used for fluidized bed solids drying. Three superficial gas velocities are investigated. The particle temperature and density are accurately described. Besides, the Sherwood number is well captured by the coarse-graining simulations.

## 1 Introduction

Solids drying is commonly carried out in a gas–solid fluidized bed. Fluidized beds have outstanding solid mixing characteristics, leading to the desired high heat and mass transfer rates. These high transfer rates are crucial for solids drying, as solids drying is a very energy-intensive process due to the high latent heat of vaporization.^1^ However, fluidized bed gas–solid contacting mechanisms are complex, resulting in enormous challenges regarding scale-up and process prediction. The computational fluid dynamics (CFD)-discrete element method (DEM) can be used to acquire more insights into fluidized bed solids drying.^2^

Fluidized bed drying is studied using CFD-DEM^3−8^ in the scientific literature. It is concluded that the studied simulation scale is generally limited due to the computationally costly discrete element method. As a consequence, the available CFD-DEM drying studies considered a relatively small number of particles. Coarse-grained CFD-DEM^9^ is a method to alleviate this limitation, giving rise to researching larger fluidized bed systems. In CFD-DEM coarse-graining simulations, the actual number of DEM particles is replaced with a smaller number of larger particles by the use of scaling laws. For a detailed overview of the available coarse-graining techniques, see the review in ref (9). One of the most extensively researched methods is presented in ref (10). Subsequently, fluidized beds were studied using this scaling law.^11−14^ Several other coarse-graining techniques exist; see, for example, refs (15−18). However, based on the review in ref (9), it is clear that research is mainly focused on bed hydrodynamics, and only a few literature studies reported coarse-graining scaling laws for gas-particle heat and mass transfer.

The first scientific work about coarse-graining heat transfer was published by ref (19). Recently, ref (20) used CFD-DEM coarse-graining for a spray coating process, refs (21), (22) employed coarse-graining for biomass gasification research, whereas gas–solid heat transfer was investigated in our previous work.^23^ Reference (24) studied coarse-graining CFD-DEM fluidized bed drying by applying coarse-graining techniques for fluidized bed gas–solid heat transfer. Scaled moisture transport was not incorporated into their work, and their original system contained only *O*(10^4^) particles.

In this study, coarse-graining CFD-DEM fluidized bed drying is studied for a larger number of DEM particles (504 000), allowing one to compare the coarse-graining drying behavior of a larger fluidized bed. The coarse-graining CFD-DEM model of ref (10) used in our previous works (see refs (25) and (23)) is extended to solids drying. The CFD-DEM model is based on the combination of the CFD code FoxBerry^26^ with the DEM software MercuryDPM.^27^ The CFD-DEM code has been validated and verified in our previous works.

In the article, we present coarse-graining CFD-DEM modeling methods, including solid drying, in the next section. Subsequently, Section 3 provides the fluidized bed simulation setup parameters. The simulation results are discussed in Section 4, followed by a final conclusion on the suitability of the solid drying scaling method, which is given in Section 5.

## 2 Modeling Method

The gas phase is modeled using CFD, governed by the continuity equation and volume-averaged Navier–Stokes equations. The gas-phase density is calculated using the ideal gas law, given by
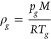
1

The molecular weight of the gas is assumed to be constant due to the relatively low moisture mass fraction. **S** is the gas-particle momentum source term, given by eq 2. Furthermore, the drag correlation of ref (28) is used in our work. For more information about the momentum source term, see refs (25) and (23).
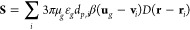
2

The thermal energy balance is shown in eq 3
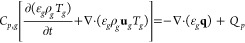
3

Using
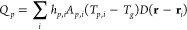
4*h*_*p*,*i*_ is obtained using the correlation of ref (29), given by
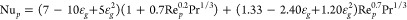
5using
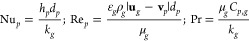
6

The heat conduction is modeled via an effective conductivity^30^
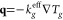
7
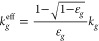
8

The gas-phase moisture mass fraction is calculated according to
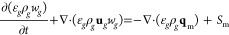
9where *S*_m_ represents the source term used for the gas-particle mass transfer
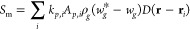
10w* is the partial vapor content at the particle surface using the water partial vapor pressure (*p*_vap_) correlation of ref (31) and the particle temperature (*T*_*p*,*i*_). See the discrete phase subsection for more information about liquid evaporation.
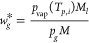
11*k*_*p*,*i*_, is obtained using the correlation of ref (29)
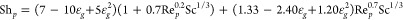
12

Using
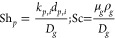
13

The effective diffusive flux in eq 9 is calculated using Fick’s law, where the effective diffusivity is computed similarly to the effective conductivity as
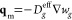
14
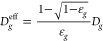
15

The particle phase is modeled by using the discrete element method. The particle mass, *m*_*i*_, is dependent on the liquid mass, see eq 17. The particle–particle and particle-wall collisions are based on the soft-sphere model originally developed by ref (32). Similar to our experimental works,^33,34^ water is contained inside the γ-Al_2_O_3_ particles; hence, no liquid bridge force model is incorporated in the model.

The heat balance for particle, *i*, is given by
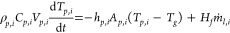
16where *h*_*p*,*i*_ is obtained via the Nusselt number; see eq 6. *H*_*f*_ is the specific latent heat of evaporation (2257 kJ/kg). ρ_*p*,*i*_ is the particle density, which is dependent on the liquid mass captured inside the solid material, calculated via
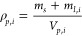
17using the dry particle (*m*_*s*_) and water (*m*_*l*_) mass. The heat capacity *C*_*p*,*i*_ is calculated according to
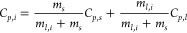
18 is the liquid evaporation rate calculated via
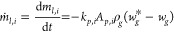
19using the partial vapor content given by eq 11. No intraparticle mass and heat transfer limitations are considered.

### 2.1 Scaling Model

The total solids mass and volume of the coarse-grained system are equal to those of the base case system (coarse-graining ratio equal to 1). In coarse-graining simulations, one particle represents *l*^3^ original particles, indicated by the coarse-graining ratio *l*. Hence, in these simulations, the particle diameter is multiplied by *l* (i.e., *d*_*p*,*c*_ = *ld*_*p*_). Therefore, the number of particles in coarse-grained simulations is reduced by a factor *l*^–3^ compared to the original system. See ref (10) and our previous works refs (25) and (23) for more information.

#### 2.1.1 Heat and Mass Transfer

As indicated, the particle mass is scaled with *l*^3^, and the liquid mass for coarse-grained particles is given by *m*_*l*,*c*,*j*_ = *l*^3^*m*_*l*,*i*_. Therefore, the right-hand side of the liquid evaporation rate, shown in eq 20, has to be scaled by *l*^3^.
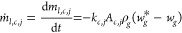
20

The area is scaled by *l*^2^. Therefore, the coarse-grained mass transfer coefficient becomes
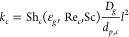
21

The coarse-grained Sherwood number uses the Reynolds number, wherein the original particle diameter is utilized. The Schmidt number is unchanged since the gas-phase properties remain constant upon coarse-graining.

The heat balance for coarse-grained particles, shown in eq 22, is also scaled by *l*^3^ due to *V*_*c*,*j*_ = *l*^3^*V*_*p*,*i*_ and *h*_*c*,*j*_*A*_*c*,*j*_ = *l*^3^*h*_*p*,*i*_*A*_*p*,*i*_ and  = . The density, specific heat capacity, and the latent heat of vaporization remain constant upon coarse-graining.
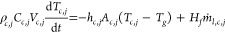
22

Using the analogy for heat and mass transfer, the Nusselt number correlation uses the Reynolds number, wherein the original particle diameter is utilized. Furthermore, the Prandtl number is constant upon coarse-graining. Therefore, the coarse-grained gas-particle heat transfer coefficient becomes
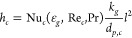
23

## 3 Simulation Setup

The solid drying was investigated in a 3D fluidized bed (0.2 × 0.035 × 0.02 m, height × width × depth). Superficial gas velocities equal to 0.30, 0.35, and 0.40 m/s were investigated. The original system uses 504 000 γ-Al_2_O_3_ particles. The DEM collision properties for γ-Al_2_O_3_ particles were obtained by ref (35). The coarse-graining method was studied by using ratios equal to 1.5, 2, and 2.5. This results in 149 333, 63 000, and 32 256 particles, respectively. The maximum studied ratio is relatively low, as a consequence of the 3D fluidized bed size. A larger 3D fluidized bed results in a base case system that is too large. The simulation parameters are given in Table 1. The solid drying is simulated for 180 s, which costs around 90 days for the base case simulations. Note that 180 s turned out to be insufficient to fully dry the solid material, but it is sufficient to draw conclusions regarding the influence of coarse-graining.

**Table 1:** Simulation Parameters Utilized for Investigating Coarse-Grain Solid Drying in a 3D Fluidized Bed^a^

parameter	symbol	value	unit
particle diameter	*d*_*p*_	**5 × 10**^**–4**^	m
inlet velocity nitrogen	*a*_0_	0.30, 0.35 and 0.40	m/s
dry particle density	ρ_*p*_	1040	kg/m^3^
dry particle heat capacity	*C*_*p*_	779	J/kg/K
water heat capacity	*C*_*p*_	4184	J/kg/K
friction coefficient	μ_0_	0.1	
normal coefficient of restitution	*e*_*n*_	0.74	
tangential coefficient of restitution	*e*_*t*_	0.1	
CFD time step	*t*_flow_	2.5 × 10^–5^	s
DEM time step	*t*_DEM_	2.5 × 10^–6^	s
time simulated	*t*	180	s
number of grid cells (width)	*N*_*x*_	28	
number of grid cells (depth)	*N*_*y*_	16	
number of grid cells (height)	*N*_*z*_	160	

a The particle diameter needs to be scaled in the coarse-graining systems, hence this value is given in bold.

In the initialization phase, wet particles with a uniform temperature of 328.15 K and a density of 1650.8 kg/m^3^ were positioned in a lattice structure. Water is contained inside the solid material. A uniform superficial velocity boundary condition at the bottom of the column was applied, where moisture-free nitrogen gas with a temperature equal to 363.15 K was injected. Moreover, a fixed pressure of 1 atm at the top of the domain and a no-slip boundary condition for the side walls were applied. No heat was lost through the side walls of the column.

## 4 Results and Discussion

### 4.1 Particle Configurations

**Figure 1:**
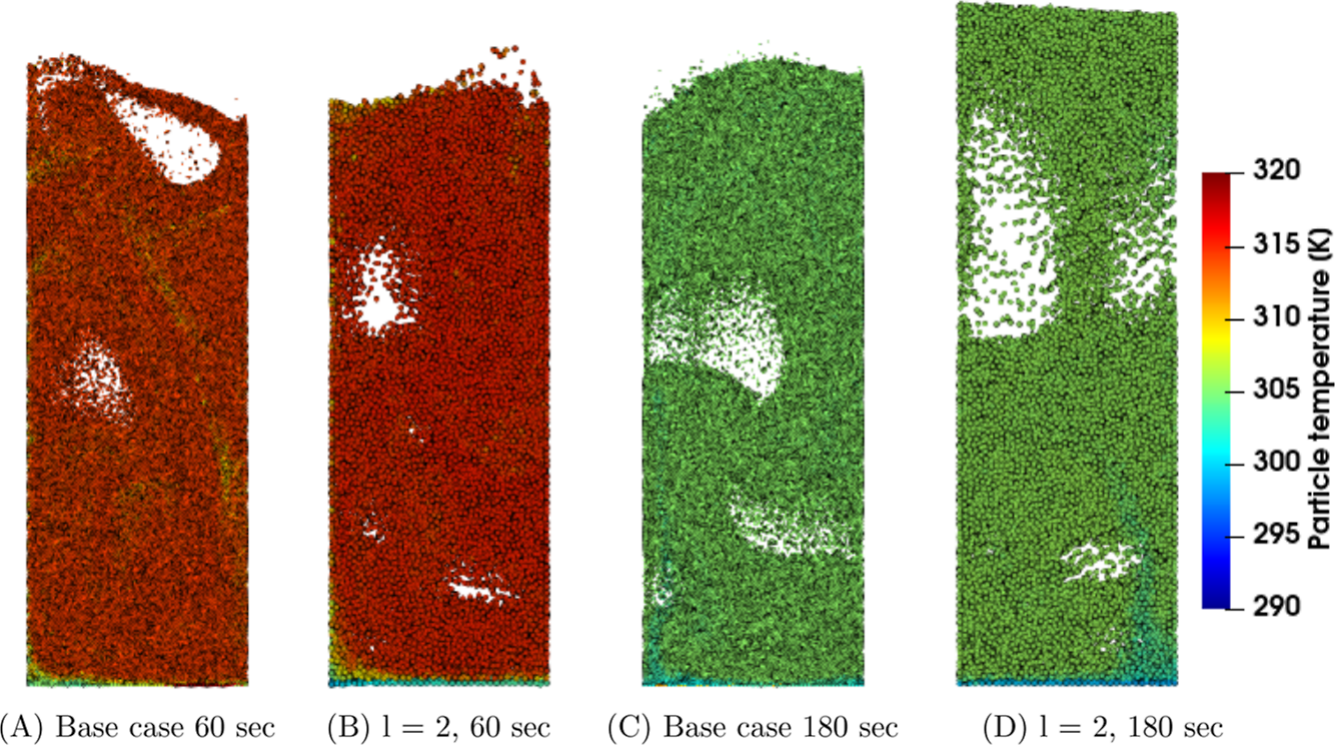
Particle temperature snapshots at the center region of the fluidized bed for the *u*_0_ = 0.30 m/s case.

**Figure 2:**
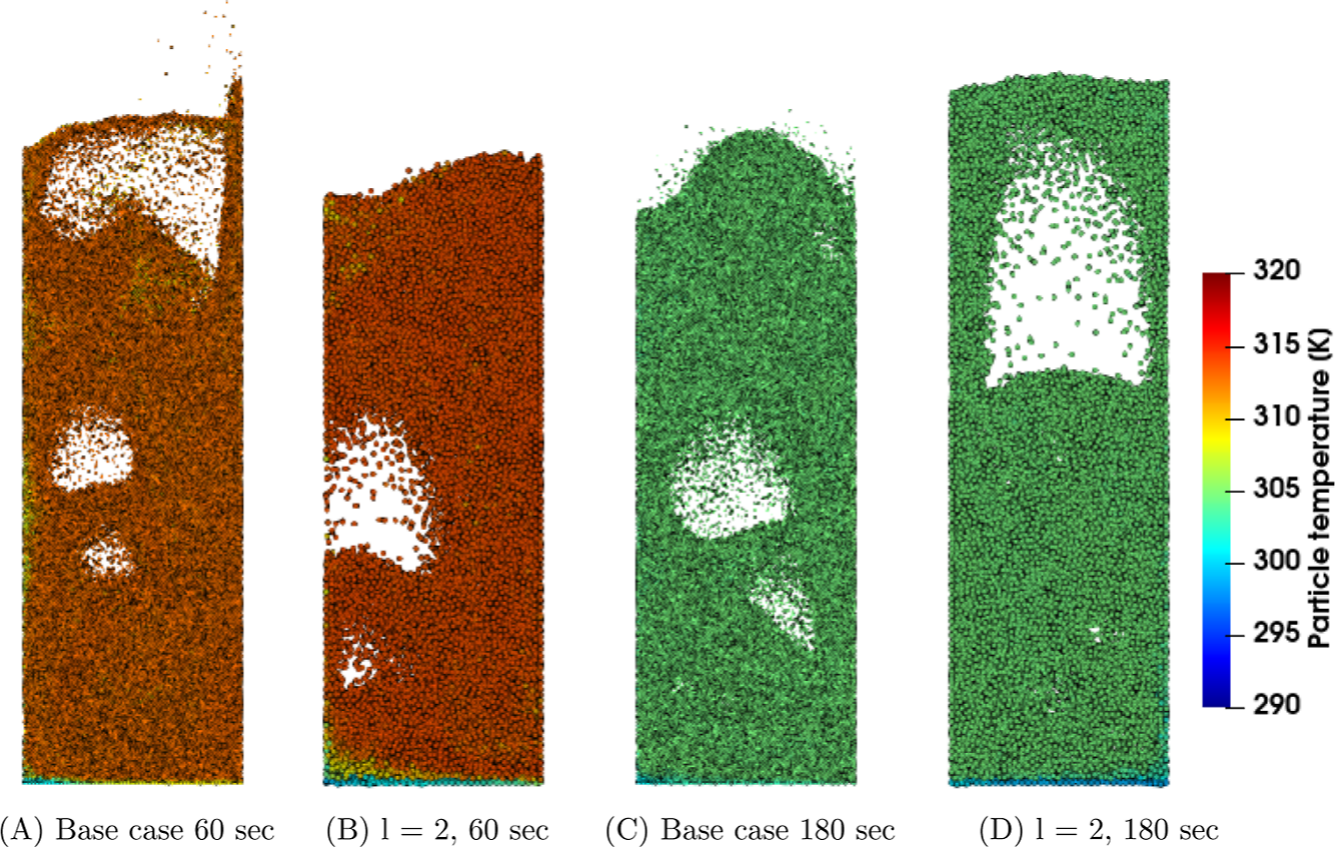
Particle temperature snapshots at the center region of the fluidized bed for the *u*_0_ = 0.35 m/s case.

**Figure 3:**
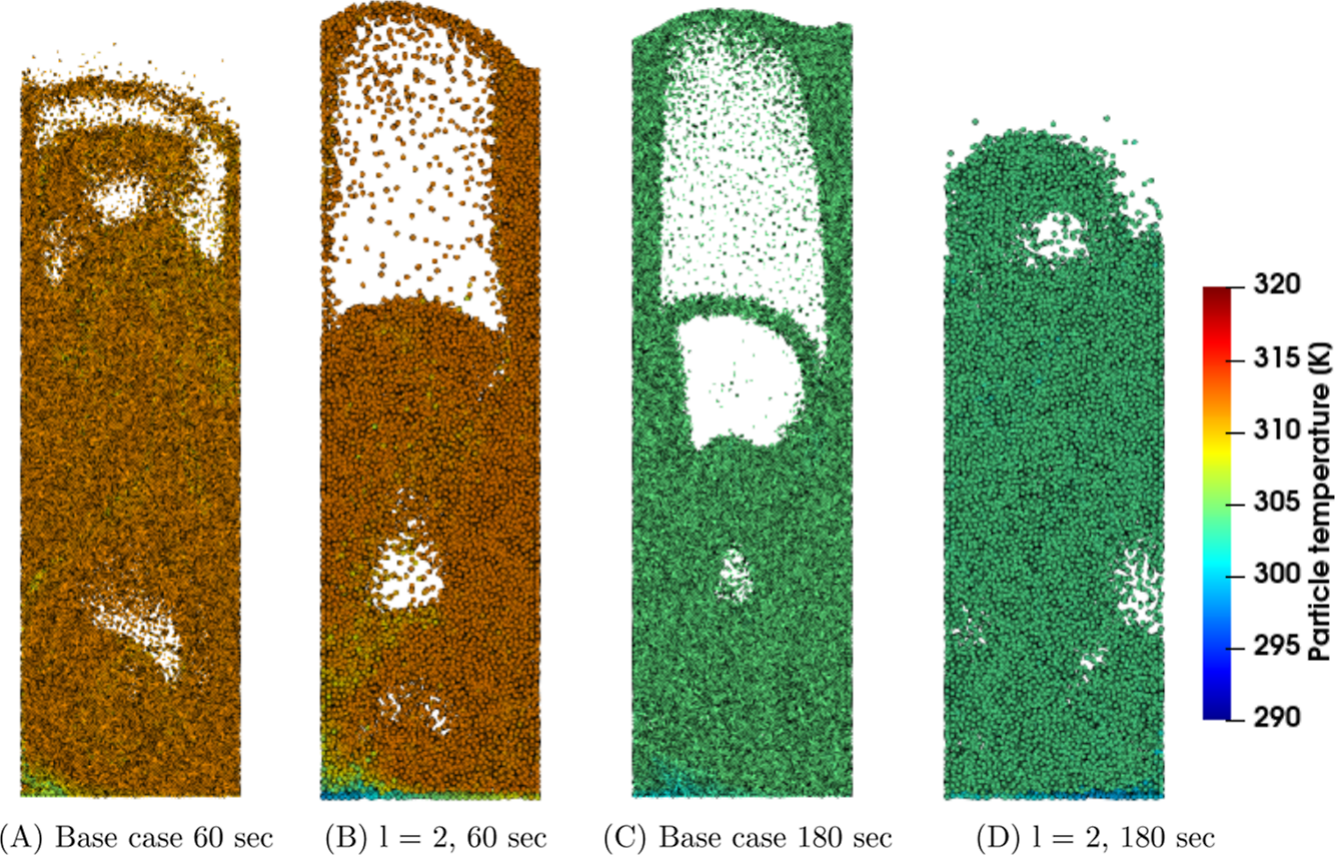
Particle temperature snapshots at the center region of the fluidized bed for the *u*_0_ = 0.40 m/s case.

First, the temperature and density in the coarse-grain simulations are analyzed. Figures 1, 2, and 3 show snapshots at the center region of the fluidized bed for the three superficial gas velocity cases, respectively. The base case is compared to the case with a coarse-grain ratio equal to 2, both at 60 and 180 s. At the start of the simulation, a uniform temperature equal to 328.15 K was applied, and due to liquid evaporation, the temperature decreased. This can be clarified by the characteristics of the drying regimes. Depending on the initial temperature, the particle temperature will decrease (in this case or increase when the wet-bulb temperature is above the initial particle temperature) until it reaches the wet-bulb temperature. Identical temperature color bars between the different cases were used to show the larger particle temperature decrease for higher volumetric gas flow rates. This means that the wet-bulb temperature is reached earlier.

Increasing the superficial gas velocity results in an increased gas bubble size since a higher gas velocity results in enhanced bubble coalescence. This trend is clearly noted by a comparison of the three cases. However, this trend is not visible in, for example, Figure 3D due to the dynamics of bubble formation, propagation, and eruption. Furthermore, larger bubbles result in a greater bed expansion. The scaling law simulations show good agreement with the original system.

**Figure 4:**
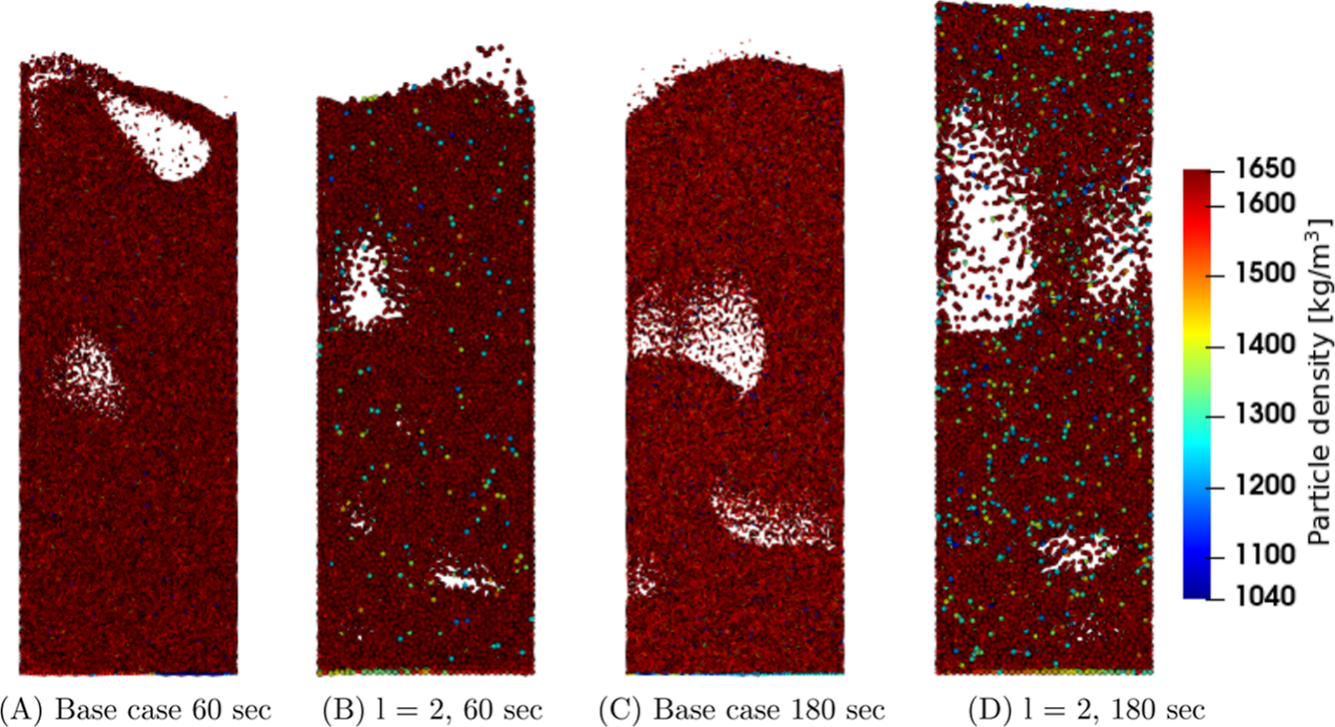
Particle density snapshots at the center region of the fluidized bed for the u_0_ = 0.30 m/s case.

**Figure 5:**
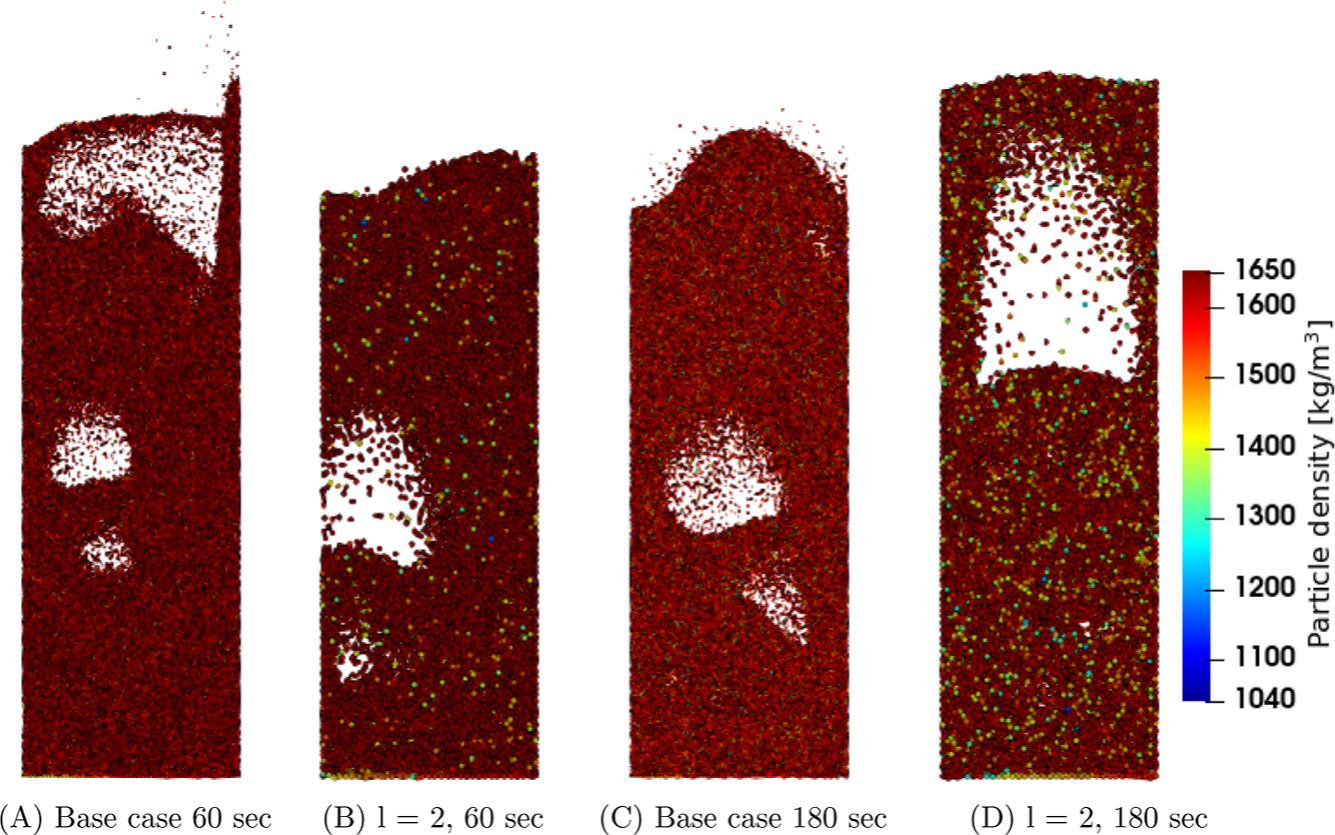
Particle density snapshots at the center region of the fluidized bed for the u_0_ = 0.35 m/s case.

**Figure 6:**
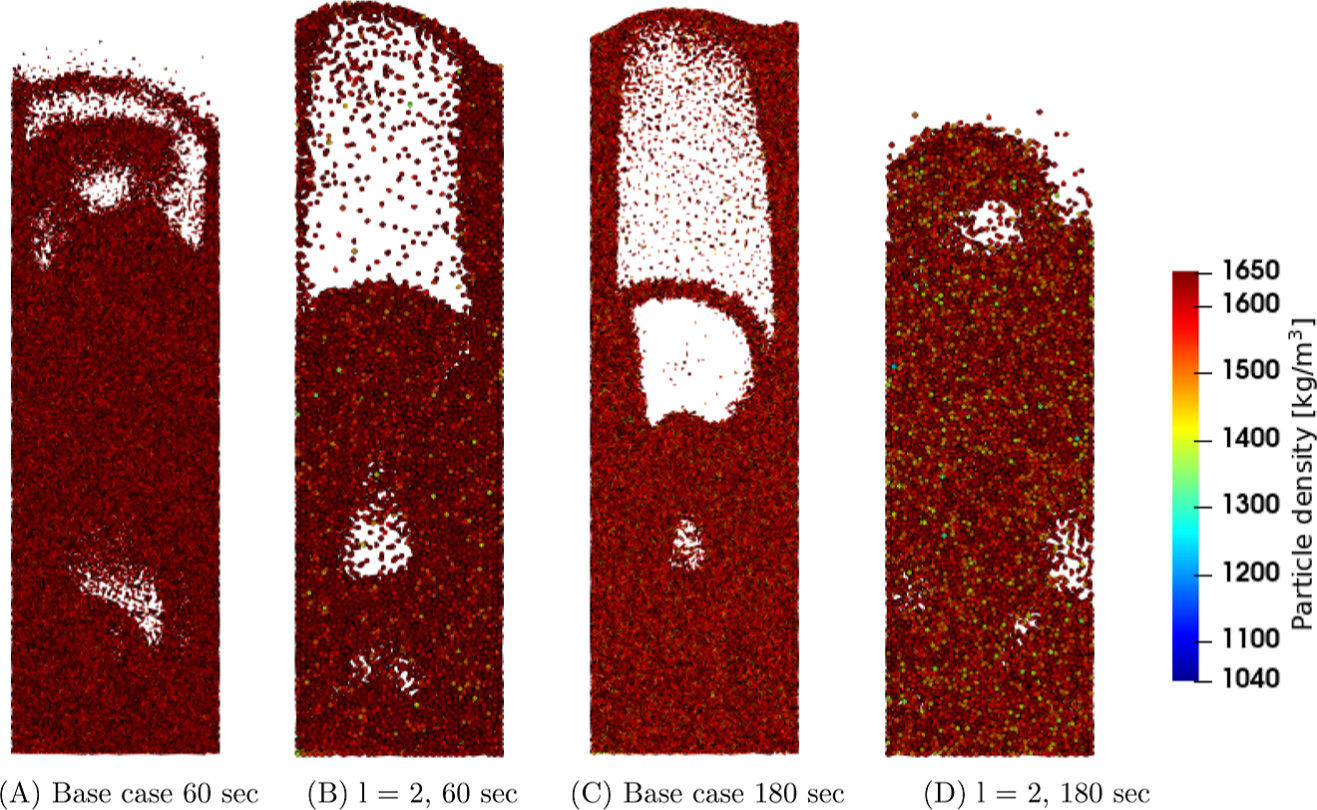
Particle density snapshots at the center region of the fluidized bed for the u_0_ = 0.40 m/s case.

Figures 4, 5, and 6 show snapshots for the *u*_0_ = 0.30, 0.35, and 0.40 m/s cases, respectively. The snapshots are taken at 60 and 180 s. In Figure 4C,D, a difference in the particle density inside the bed can be observed. In the coarse-graining system, the bed contains some randomly positioned particles possessing a relatively lower particle density compared to the base case. In the case of a higher applied superficial gas velocity, this is less pronounced (Figure 6). Therefore, it becomes interesting to investigate the effect of coarse-graining on the solids drying in more detail. This analysis is performed in the next subsections whereof the mentioned particle density differences are further investigated in Subsection 4.3.

### 4.2 Particle Temperature

**Figure 7:**
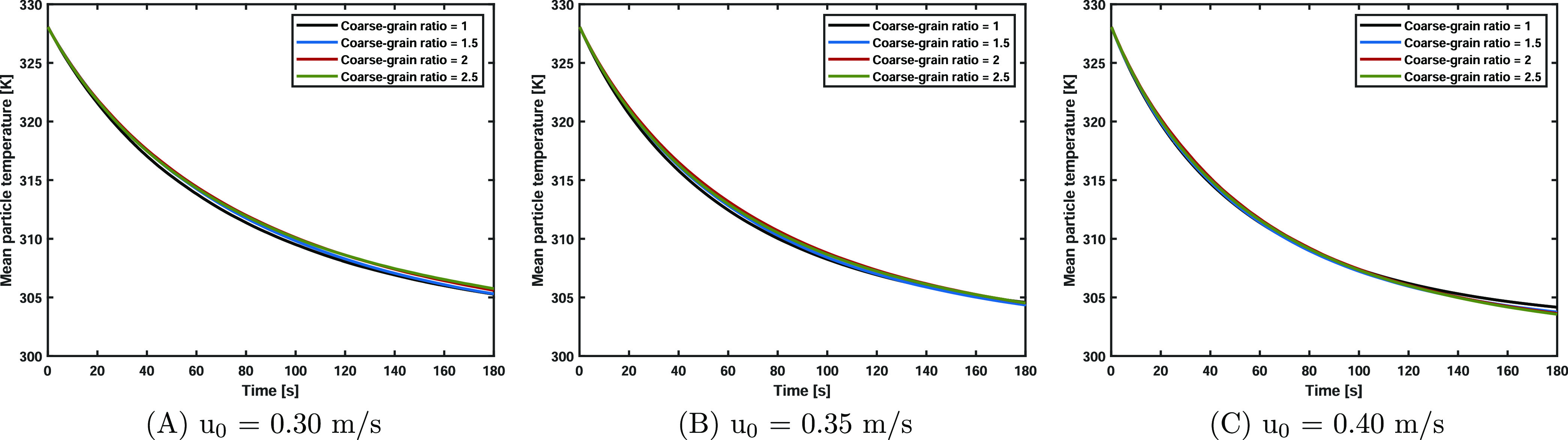
Mean particle temperature versus time for three superficial velocities. The temperature gradually reaches the wet-bulb temperature due to liquid evaporation. The coarse-grained simulations flawlessly match the average temperature over time.

Figure 7 shows the mean temperature versus time for the three superficial gas velocity cases. Please note that the grain temperature is analyzed in the coarse-graining cases. At the start of the simulation, the particle temperature was initialized at 328.15 K, and liquid evaporation resulted in a particle temperature decrease until the wet-bulb temperature was reached. Due to the computational time required for the base case simulations, the solids drying process is not fully completed toward the end of the simulation. It can be clearly seen that a higher gas volumetric flow rate leads to a steeper temperature decline due to the increased evaporation rate. Besides, it is concluded that the coarse-grained simulations flawlessly match the average temperature over time.

**Figure 8:**
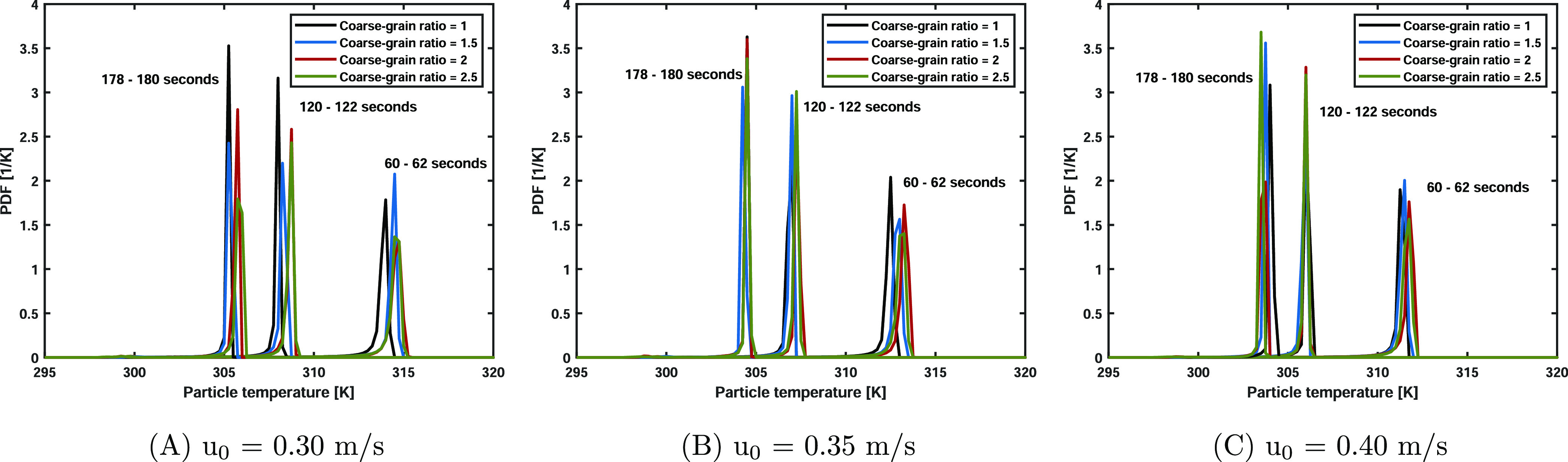
Particle temperature PDF at 60–62, 120–122, and 178–180 s. Due to liquid evaporation, the solids temperature decreases toward the wet-bulb temperature. The coarse-grained simulations show a good resemblance to the base cases.

A normalized particle temperature probability density function (PDF) shows the particle temperature distribution. Figure 8 shows the PDFs at 60–62, 120–122, and 178–180 s, respectively. In the first time range (60–62 s), all cases show a pronounced tail toward the lower temperatures. Liquid evaporation requires thermal energy, resulting in lower particle temperatures. Over time, this tail decreases in size, and the particle temperature distribution narrows. This can be explained by the lower saturation partial vapor content (eq 11) at lower temperatures. Therefore, less liquid is evaporated per unit of time, resulting in a lower evaporation energy contribution to the particle thermal energy balance and less pronounced temperature tails. Comparable to the average temperature, coarse-graining simulations accurately predict the original systems. Nevertheless, small differences are discernible. This can be explained by the effects arising mainly in the bottom region of the bed (see also our subsequent analysis). In this region, a high evaporation rate occurs as hot and unsaturated gas is injected into the bottom of the column. Before leaving the bed into the freeboard, the gas is fully saturated, resulting in a coinciding average particle temperature. However, a difference in the local energy transfer inside the bed is found in the coarse-graining simulations, which leads to these small discrepancies noted in the PDFs. These differences are less profound by increasing the superficial gas velocity, and we will further discuss these discrepancies in the next paragraph.

**Figure 9:**
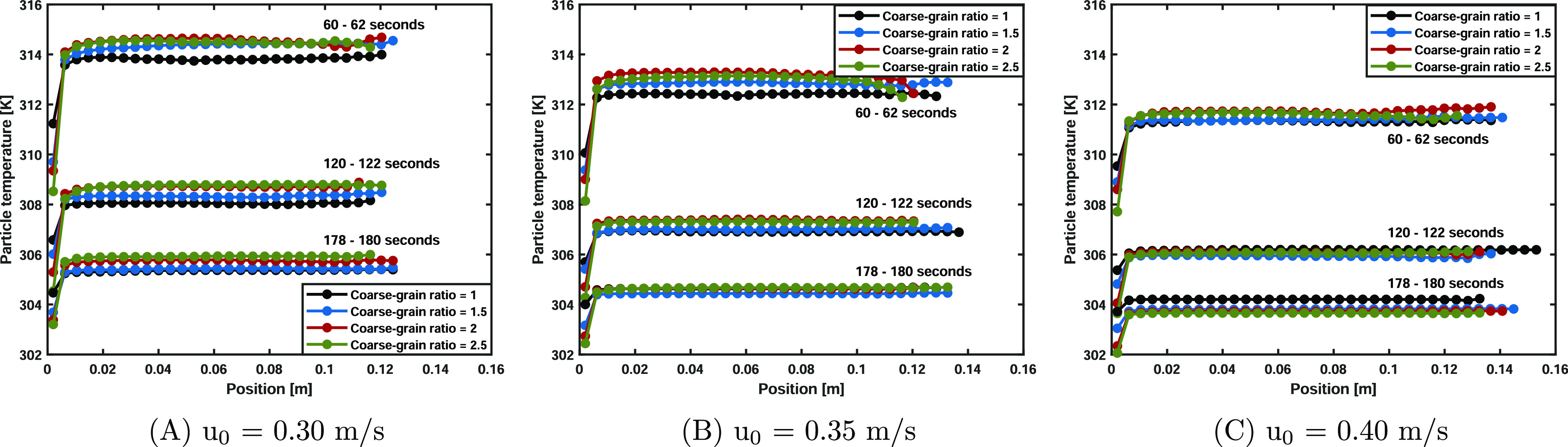
Particle temperature over the axial position. The coarse-grained simulations show a good resemblance with the base cases.

The local particle temperature discrepancies can be also explained by analyzing the axial temperature profiles, as shown in Figure 9. It can be clearly seen that an increased bed expansion is displayed with increasing superficial gas velocity. In the bottom region, low-temperature regions are observed, which are caused by liquid evaporation. Over time, these temperature zones decrease in size due to the lower saturation partial vapor content (eq 11) at lower temperatures. It is well known that an increased superficial gas velocity results in more vigorous bed mixing and a higher solids circulation rate. Besides, it also results in more liquid evaporation per unit of time. Based on the temperature profiles observed in Figure 9, the increased solid mixing rate at higher superficial gas velocities is more dominant compared to the increased evaporation rate in terms of bed homogeneity as the low-temperature tails decrease in size. As reported by ref (23), in coarse-graining simulations, the particles residing in the bottom region are relatively colder, whereas the solids located up in the bed possess a somewhat higher temperature. By increasing the superficial gas velocity, the differences become less pronounced and a better correspondence is observed; this is also in accordance with the particle temperature PDFs. The main reason for the discrepancies can be found in the high inlet temperature. This creates a large gas-particle temperature driving force, which is quickly reduced due to the fast energy transfer with the solids located in the bottom region. This results in a relatively steep decrease of the gas temperature at the inlet, and in the coarse-graining simulations, this fast reduction is not well captured due to the averaging nature since the local details of the original system are not resolved.

### 4.3 Particle Density

**Figure 10:**
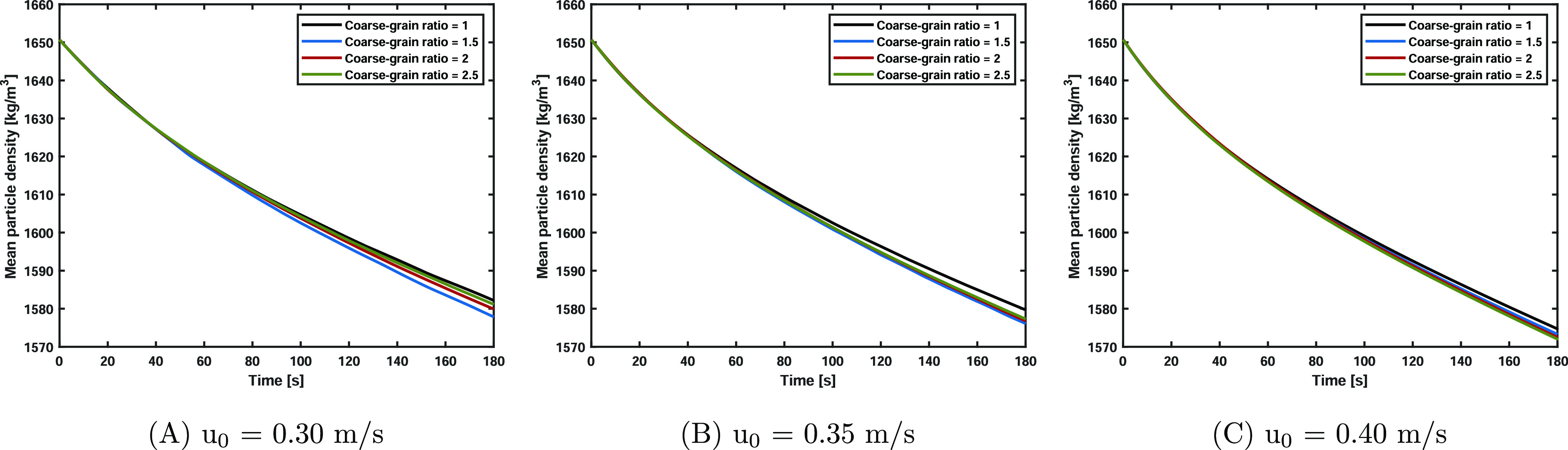
Mean particle density versus time. The particle density is reduced due to liquid evaporation. The scaling law accurately describes the original mean particle density.

The mean particle density versus time is shown in Figure 10. Initially, the particle density is equal to 1650.8 kg/m^3^. Please note that the grain density is analyzed in coarse-grained simulations. Due to liquid evaporation, the particle density decreases over time. Increasing the superficial gas velocity results in more liquid evaporation per unit of time. The coarse-grained simulations describe the time evolution of the mean particle density well. This is also expected based on the previous discussion about the particle temperature, as the liquid evaporation rate is highly dependent on particle temperature, which itself shows good correspondence with the original, noncoarse-grained, simulation.

**Figure 11:**
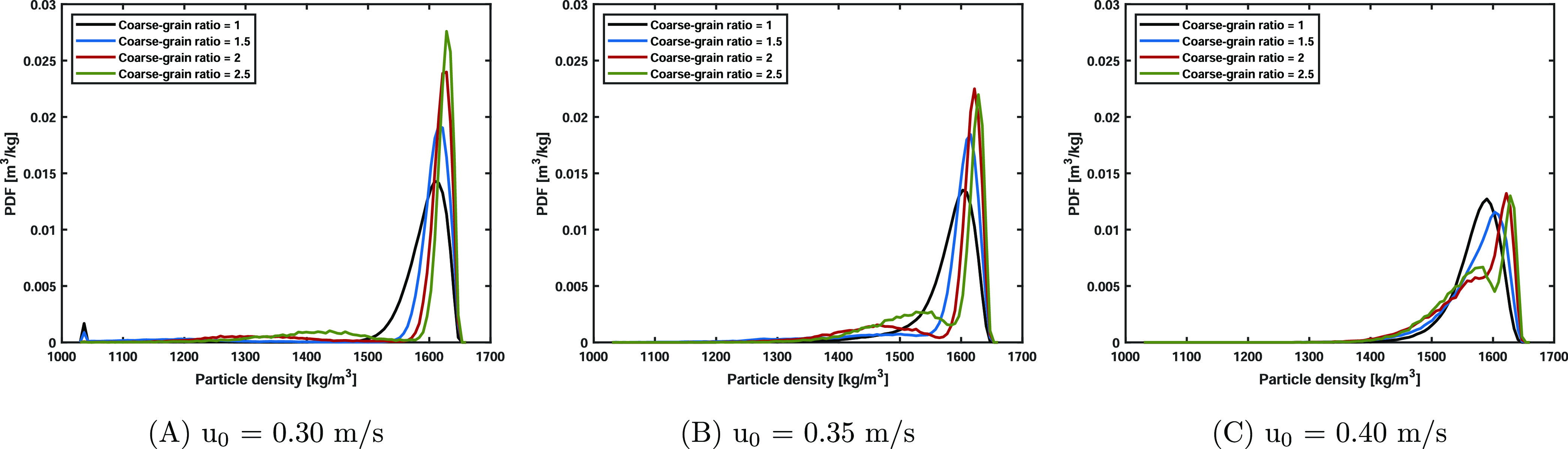
Particle density PDF using the particle densities in the time span of 178–180 s for the three studied superficial gas velocities. Deviations due to the coarse-graining averaging are observed in the particle density distributions.

The normalized particle density PDF using the particle densities in the time span of 178–180 s is shown in Figure 11. Due to the liquid evaporation, PDF tails extending into the lower density region are observed throughout all simulations. However, a remarkable effect based on the superficial gas velocity is noted, as in the lowest case, a small peak of fully dried material (ρ = 1040 kg/m^3^) and a relatively large peak around 1600 kg/m^3^ are observed. The peak, indicating fully dried material, is not observed at higher superficial gas velocity cases and therefore clearly correlated to the solids mixing rate.

The coarse-grained systems show discrepancies compared with the original systems. In the lowest superficial gas velocity case, relatively sharper peaks, increasing upon coarse-graining ratio, around 1600 kg/m^3^ are observed. Besides, the dry density peak decreases with the level of coarse-graining, and a small and broad peak ranging from 1200 to 1500 kg/m^3^ appears. Both are mainly caused by the differences occurring in the bottom region as also observed in the particle temperature analysis. In the other two superficial gas velocity cases (*u*_0_ = 0.35 and 0.40 m/s), two distinctive peaks are noted in the coarse-grained simulations, while in the original systems, one broad peak is observed. Similar to the lowest velocity case, this is a consequence of the averaging nature of coarse-graining. However, the lower density peak is shifted toward the right side due to the more intense solids circulation rate.

**Figure 12:**
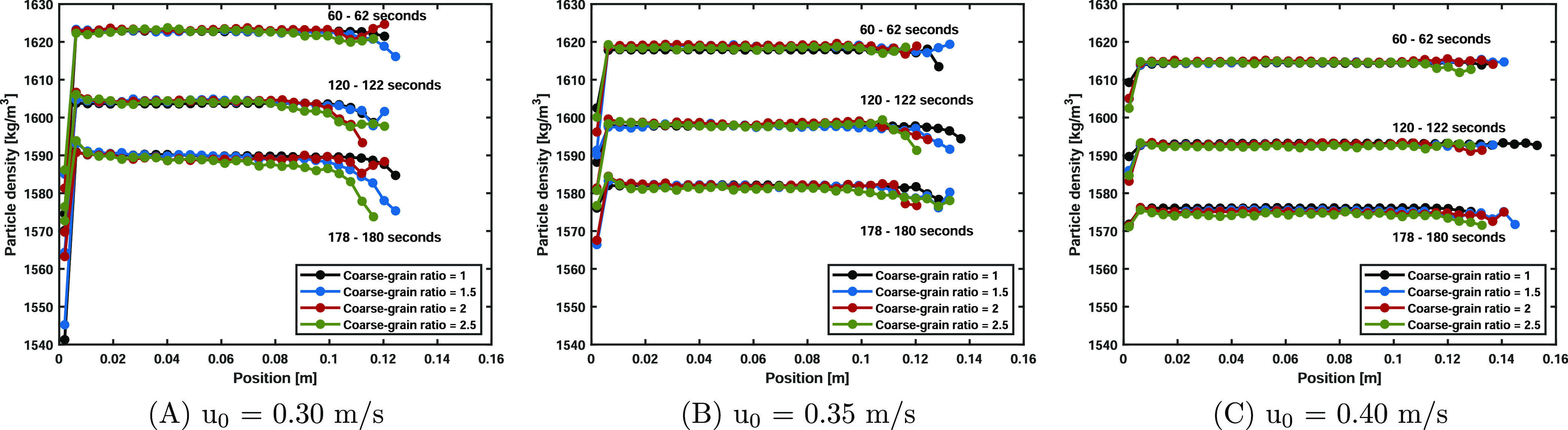
Particle density over the axial position for the three studied superficial velocities (*u*_0_ = 0.30, 0.35, and 0.40 m/s). The coarse-graining scaling law shows good correspondence with the original system.

The particle density over the axial position shown in Figure 12 substantiates the observed differences in the PDFs. It can be clearly seen that increasing the superficial gas velocity results in smaller density drops in the bottom region of the bed due to the intensified solids mixing. This corresponds with the discussion regarding particle temperatures in the previous paragraph, where we concluded that the increased solids mixing rate at higher superficial gas velocities is more dominant compared to the increased evaporation rate. In the lowest superficial gas velocity case, the coarse-graining cases show a relatively smaller density drop in the bottom region, caused by similar effects as discussed in the particle temperature analysis. The larger superficial gas velocities result in better solids mixing; therefore, the contact time with the unsaturated gas in the bottom region is lower, leading to smaller density drops and a better correspondence with the original system.

### 4.4 Gas-Particle Sherwood Number

**Figure 13:**
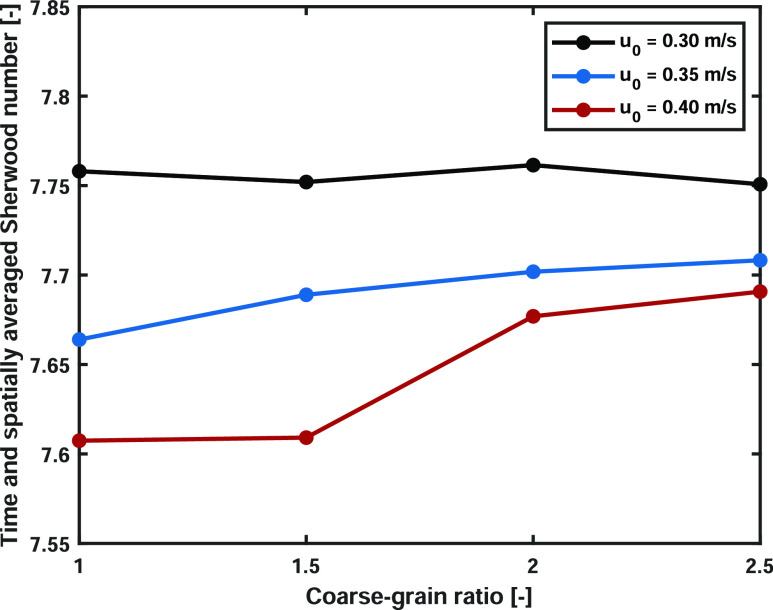
Time and spatially averaged Sherwood number of the three different superficial gas velocities over the coarse-graining ratio.

In the previous subsections, the temperature and density were analyzed. The particle drying is also heavily dependent on the transfer rates, which should be accurately described by the scaling law. The gas-particle heat and mass transfer coefficients are computed using the Gunn correlation (see eqs 5 and 12 respectively). In this section, only the Sherwood number will be discussed. The time and spatially averaged Sherwood number is shown in Figure 13. The first 2.5 s were not incorporated in order to exclude startup effects. It is clearly observed that the average Sherwood number is lower at higher superficial gas velocities, which is in correspondence with the analysis of the Nusselt number shown in the literature.^23,36,37^ For the scaling law simulations, the obtained Sherwood numbers are slightly altered, with a maximum difference of approximately 1%. Therefore, the scaling law is capable of perfectly predicting the average Sherwood number of the base case.

**Figure 14:**
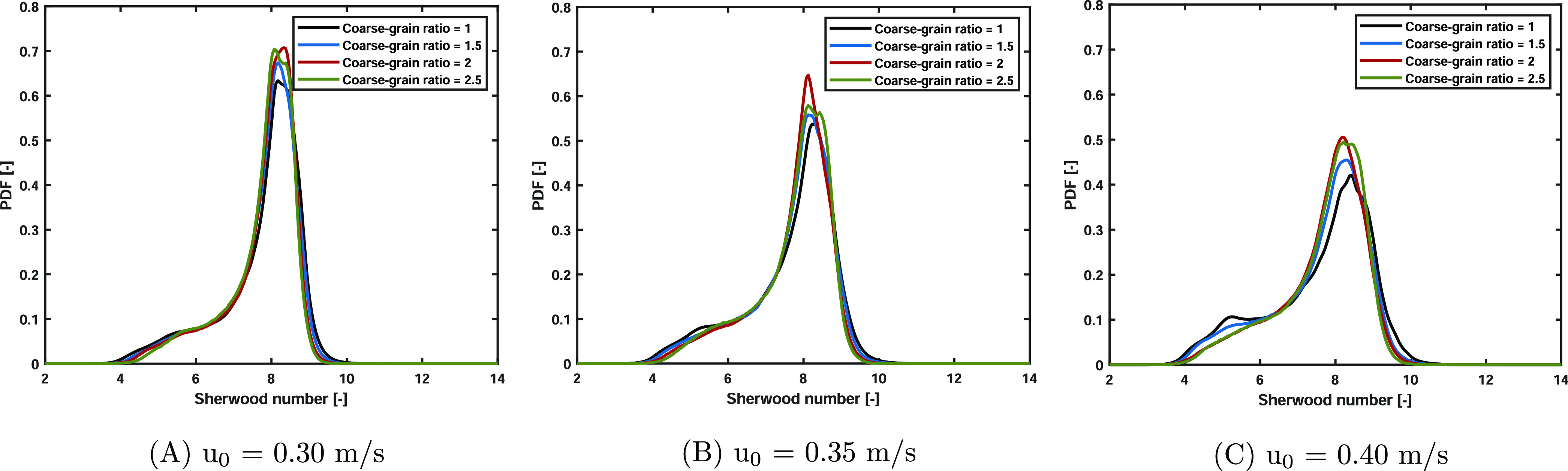
Normalized Sherwood number probability density functions for the three studied superficial gas velocity cases. The Sherwood numbers obtained in the coarse-grained simulations perfectly match those in the base case PDFs.

The effect of the scaling law on the Sherwood number can be described in more detail by the time and spatial probability density functions shown in Figure 14. Wider Sherwood number distributions are obtained at higher superficial gas velocities. This can be related to the intensified bubbling behavior. The coarse-grained simulations also result in these wider Sherwood number distributions at increased superficial gas velocities. However, it is noted that slightly more deviations are observed when the superficial gas velocity is increased. As discussed in refs (23), (36) for heat transfer, high Nusselt numbers are found in the bubble wake. On the contrary, low values are observed in the bubble clouds. Coarse-graining is an averaging method; hence, the bubble wake and cloud regions are less pronounced, resulting in more narrow distributions of the Sherwood number. At lower gas velocities, the bed is in a homogeneous state, and relatively small bubbles appear. Hence, this described effect is less pronounced, and smaller differences in the PDF of the Sherwood number are obtained.

## 5 Conclusions

Coarse-grained CFD-DEM is extended to fluidized bed drying. Coarse-grained fluidized bed drying was investigated using three superficial gas velocities equal to 0.30, 0.35, and 0.40 m/s. The coarse-grain scaling law led to a good description of the average particle temperature and density. However, small differences in the particle temperature and density probability functions were obtained. These deviations were mainly caused by the averaging effects of coarse-graining, resulting in differences in the bottom region of the bed, where steep profiles of particle temperature and density were encountered. These differences are less pronounced for higher superficial gas velocities due to more intense solids mixing.

Furthermore, gas-particle mass transfer was investigated via the Sherwood number. The time and spatially averaged Sherwood numbers were accurately predicted by the applied scaling law with a maximum deviation equal to 1%. The normalized Sherwood number probability density functions showed a good match with the base case simulations. However, slightly more peaked Sherwood distributions compared to the original systems were obtained due to the averaging nature.

## Nomenclature

*A*_*p*_: particle surface area (m^2^)
*C*_*p*_: specific heat capacity (J/kg/K)
*D*_*g*_: diffusivity (m^2^/s)
*d*_*p*,*i*_: particle diameter (m)
*e*_*n*_: normal restitution coefficient (−)
*e*_*t*_: tangential restitution coefficient (−)
**g**: gravitational acceleration (m/s^2^)
*h*: heat transfer coefficient (W/m^2^/K)
*H*_*f*_: latent heat of vaporization (kJ/kg)
*k*: mass transfer coefficient (m/s)
*k*: thermal conductivity (W/m/K)
*l*: coarse-graining ratio (−)
*m*_*i*_: particle mass (kg)
*M*_*l*_: water molecular weight (kg/mol)
Nu: Nusselt number (−)
*p*_*g*_: gas pressure (Pa)
Pr: Prandtl number (−)
*Q*_*p*_: heat source term (W/m^3^)
*R*: gas constant (J/K/mol)
Re: Reynolds number (−)
*S*_*m*_: mass source term (kg/m^3^/s)
Sc: Schmidt number (−)
Sh: Sherwood number (−)
*T*: temperature (K)
*t*: time (s)
**u**_*g*_: gas velocity (m/s)
**r**_*i*_: particle position (m)
**v**_*i*_: particle velocity (m/s)
*V*_*i*_: particle volume (m^3^)
*w*: moisture mass fraction (kg/kg)
*w**: partial vapor content (kg/kg)

## Greek letters

ε_*g*_: gas holdup (−)
ρ: density (kg/m^3^)

## Sub/superscripts

*c*, *j*: individual coarse-grained particle
*eff*: effective property
*g*: gas property
*i*: individual particle
*l*: liquid
*p*: particle property

## Abbreviations

CFD: computational fluid dynamics
DEM: discrete element method
